# Pulmonary Arterial Hypertension Secondary to Ethanol Sclerotherapy for Renal Artery Embolization

**DOI:** 10.1155/2014/452452

**Published:** 2014-06-15

**Authors:** Raya Saba, James Davis, Arvind Balavenkataraman, Aibek E. Mirrakhimov, Aram Barbaryan, Shyam Chalise, Nkemakolam Iroegbu

**Affiliations:** Saint Joseph Hospital, Department of Internal Medicine, 2900 N Lakeshore Drive, Chicago, IL 60657, USA

## Abstract

Pulmonary arterial hypertension (PAH) has been reported as a major complication to consider and promptly manage in the use of ethanol sclerotherapy. Most of the available data on the development of PAH is derived from the use of ethanol for embolization of arteriovenous malformation, but it has been rarely reported in its other fields of application. We describe a case of outpatient renal artery embolization using ethanol, in which respiratory failure develops secondary to PAH despite adhering to safe practice protocols. We highlight the importance of pulmonary arterial pressure monitoring and the treatment steps to follow in order to avoid irreversible fatal outcomes.

## 1. Introduction

Ethanol is currently the primary choice for vessel embolization. Ethanol has a unique mechanism of action through the denaturation of cellular proteins. This results in erosion of the endothelium, permanent damage to the vascular wall, and obliteration of its lumen. These changes are largely irreversible and do not allow for the recanalization of the damaged vessels [1]. In effect, these changes render ethanol one of the most effective agents used in sclerotherapy [2, 3].

## 2. Case Description

A 73-year-old Hispanic male with a history of coronary artery disease, hypothyroidism, and arthritis underwent a CT scan of the abdomen and pelvis for an unrelated complaint and was incidentally found to have a hypervascular left renal mass suspicious for malignancy. He was referred to an outpatient percutaneous transcatheter renal artery embolization, in preparation for a subsequent radical left nephrectomy with a view to decrease intraoperative bleeding at surgery. Physical examination prior to the procedure was essentially normal and did not reveal any cardiopulmonary abnormalities. The patient received intravenous conscious sedation over a period of 60 minutes consisting of 100 mcg fentanyl and 1.0 mg midazolam, administered in intermittent doses, while undergoing continuous physiologic monitoring. Per protocol [3], the procedure was performed using a total of 40 mL of pure ethanol divided into three doses and administered in slow pushes at 15-minute intervals. This dose was derived based on a weight of roughly 80 kg, using the lower end of the recommended dose of 0.5–1 mL/kg. The result was a significant reduction in tumor vascularity, with approximately 10% residual renal artery tumor perfusion.

After the third ethanol injection, the patient developed cough, agitation, and acute respiratory distress with hypoxemia (SpO2 of 80%; normal >92%). He required sedation and emergent intubation for airway protection. Given the concern for pulmonary embolism, pulmonary angiography was immediately performed and showed patent pulmonary arteries, without central or segmental pulmonary artery embolus or vessel occlusion. The procedure confirmed the presence of PAH with a mean pulmonary artery pressure (mPAP) of 70 mmHg (normal <25 mmHg). Acute pulmonary vasospasm due to venous shunting from the systemic intra-arterial ethanol administration was suspected. A Fogarty balloon was inflated in the renal artery to prevent further dissemination of ethanol into the systemic circulation. Nitroglycerin was administered directly into the pulmonary artery circulation at a rate of 5 mcg/min with a total dose of 1400 mcg; this resulted in reduction of mPAP from 70 mmHg to 30 mmHg. A pulmonary artery catheter was left in place for continued pulmonary artery pressure monitoring.

The patient was admitted to the intensive care unit for close monitoring. He was started on intravenous dexmedetomidine for sedation and analgesia. On physical examination, his vital signs were within normal limits, with no focal findings. Laboratory workup revealed mild leukocytosis (white blood cell count 11.4 k/mm cu; normal 4–11 k/mm cu), anemia (hemoglobin 8.3 g/dL; normal 13.5–17 g/dL), and elevated blood alcohol level (80 mg/dL; normal 0–20 mg/dL). There was no evidence of hemolysis, as the hemoglobin level was close to baseline and the bilirubin was normal. Electrocardiogram and cardiac enzymes were negative for acute ischemia; chest X-ray (CXR) did not show any pleural effusion, consolidation, or acute pathology; transthoracic echocardiogram (TTE) showed normal cardiac function; lower extremity ultrasound Doppler ruled out deep venous thrombosis (DVT), and recent pulmonary functions tests (PFT) showed no abnormalities in baseline respiratory function. Arterial blood gases were within normal limits and the patient was successfully weaned and extubated the following day. Pulmonary artery pressure throughout his stay stabilized at a mean of 30 mmHg before the pulmonary artery catheter was discontinued. His respiratory status remained stable and he was gradually weaned off supplemental oxygen altogether. There was no evidence of new cardiopulmonary abnormalities at the time of discharge.

## 3. Discussion

Data on ethanol sclerotherapy is largely based on its use in the treatment of arteriovenous malformations (AVMs) [4]. Although still considered first line therapy for AVMs, ethanol has a wide range of side effects as well. Its complications include, but are not limited to, skin and tissue necrosis, motor and sensory nerve damage, hemoglobulinuria, deep venous thrombosis, pulmonary embolism, PAH, and cardiac collapse [5].

Of the major complications, PAH is likely the most studied, due to its significance and not uncommon occurrence [6–9]. The hypothesized mechanism is ethanol-induced vasospasm at the precapillary level and a subsequent increase in pulmonary artery pressure. Rarely, PAH may in turn lead to right ventricular failure due to increased afterload and a subsequent left ventricular failure due to decreased preload, eventually culminating in full cardiopulmonary collapse [10]. Considering the rarity of complete cardiovascular collapse, some authors suggest that other unclear factors or mechanisms may be implicated in this fatal outcome as well [7]. These include thrombosis of small PA branches, embolization of the damaged vasculature, and idiopathic anaphylactic reactions [8].

Several researchers have examined the possible predictors and variables that may be associated with the development of PAH during ethanol sclerotherapy, in an attempt to establish a safe practice protocol. They found a positive correlation between PAH and serum ethanol level which is directly related to the amount of ethanol administered during any given procedure [11]. However, the correlation was stronger with the single injection dose than with the total cumulative dose [8].

In the absence of large confirmatory studies and clear guidelines, most practitioners continue to follow the protocol used by Yakes et al. [3]. The standard of care is to give small repetitive doses of ethanol separated by 10–15-minute intervals for a maximum total dose of 0.5–1 mL/kg of body weight. Continuous monitoring of PAP with Swan-Ganz catheter placement is recommended during the procedure and in the recovery room where delayed reactions may occur [12]. If measured PA pressure starts to increase, ethanol injection should be stopped and infusion of nitroglycerin through the Swan-Ganz catheter should be started. Alternatives to nitroglycerin include adenosine, prostaglandin E1, and inhaled nitric oxide [13]. Emergent intubation and mechanical ventilation may be indicated.

As mentioned earlier, most of the available data come from studies on the treatment of AVMs. However, the use of ethanol sclerotherapy has been expanding to involve other areas of medical treatment such as renal artery embolization (RAE). As opposed to some cases of AVMs, RAE is a less invasive procedure that is often performed in an outpatient setting. Some of its major applications include preoperative ablation of renal tumors, palliation of inoperable renal malignancies, and treatment of renal vascular malformations or massive hemorrhages [14]. Although side effects have been reported, they mainly consist of inadvertent embolization of untargeted vessels or postoperative symptoms of nausea, vomiting, and flank pain which is also known as “postinfarction syndrome” [15]. The fact that major systemic complications are rare [16] might be due to the more localized field of operation in RAE, as compared to AVMs, which may have a larger volume and allow easier access of ethanol to the systemic circulation.

Our case highlights the potential for significant adverse effects, even with minimally invasive RAE procedures. A high index of suspicion is warranted in such cases, and prompt treatment is the key. When respiratory distress develops, adequate ventilation should be provided, ethanol injection stopped, and vasodilators administered. Continuous monitoring of PAP during the procedure and in the recovery period is indicated, and its routine use may be helpful in early diagnosis and management of elevated PAP.

## 4. Conclusion

- Pulmonary artery hypertension (PAH) is a major complication of ethanol sclerotherapy and is thought to be secondary to precapillary vasospasm.
- PAH has been mostly reported in the use of ethanol sclerotherapy for treatment of arteriovenous malformations (AVMs) but may occur in minor outpatient procedures, such as preoperative renal artery embolization (RAE).
- Treatment of PAH related to ethanol sclerotherapy consists of prompt diagnosis, adequate oxygenation, administration of vasodilators, and close monitoring, preferably in the intensive care unit.
- Preventive measures include following safe practice protocols for ethanol sclerotherapy, adequate sedation and analgesia, and continuous monitoring of pulmonary artery pressure (PAP) during the procedure and in the recovery room.
- Further research is needed regarding the possible relative and absolute contraindications to ethanol sclerotherapy, such as in cases of preexisting cardiopulmonary disease.
